# Spatially patterned cytoskeletal organization shapes astrocyte branch complexity

**DOI:** 10.1101/2025.10.10.681734

**Published:** 2025-10-11

**Authors:** Meghan E. Wynne, William E. Barclay, Dharshini Gopal, Johanna J. Bergstrom, Lana T. Ho, Eva A. Lopez, Eva Nogales, Meng-meng Fu

**Affiliations:** 1. Department of Molecular and Cell Biology, University of California, Berkeley, Berkeley, California, USA; 2. National Institute of Neurological Disorders and Stroke (NINDS), National Institutes of Health (NIH), Bethesda, Maryland, USA; 3. California Institute for Quantitative Biosciences (QB3), University of California, Berkeley, Berkeley, CA, USA; 4. Howard Hughes Medical Institute, University of California, Berkeley, Berkeley, CA, USA; 5. Molecular Biophysics and Integrated Bioimaging Division, Lawrence Berkeley National Laboratory, Berkeley, CA, USA

## Abstract

Astrocytes, the most abundant cells in the brain, extend elaborate branches that enable diverse functions, from synapse maintenance to blood-brain-barrier integrity. The cytoskeletal basis of this architecture has remained unclear, since traditional culturing methods produce minimal branching. Using immunopanning and serum-free conditions, we generated primary rodent astrocytes with *in-vivo*–like morphology and surveyed their cytoskeleton using confocal microscopy and cryogenic electron tomography. We show that astrocyte microtubules are oriented primarily plus-ends–out. Proximally, microtubules appear stabilized by post-translational modifications (PTMs) and microtubule inner proteins (MIPs). Distal regions lack stabilizing microtubule PTMs, and are enriched in intermediate filament (IF) GFAP. Additionally, diverse actin microstructures, including reticular webbing, extend astrocyte boundaries beyond the microtubule–IF framework. Our results uncover fundamental principles of astrocyte cytoskeletal organization that underlie their intricate branching.

## Introduction

Astrocytes, the most abundant cell type in the brain, perform essential roles in neurotransmission, synaptogenesis, synaptic pruning, maintenance of the blood-brain barrier (BBB), and inflammatory signaling (1-4). These wide-ranging functions rely on their structurally complex and highly branched morphology, which enables individual astrocytes to simultaneously interact with diverse cell types, such as neurons, vascular cells, oligodendrocytes, and microglia (5, 6). Astrocyte morphology also dynamically changes in response to environmental cues (7, 8), transitioning from a highly-branched homeostatic state to an amoeboid reactive state with hypertrophy of branches following injury or disease (8-10). Although the structural complexity of astrocytes underlies their functions, the cytoskeletal organization that drives the formation, maintenance, and plasticity of astrocyte branches remains obscure (6, 11).

An outstanding question in astrocyte cell biology is the polarity of microtubules (11). Microtubules, which serve as tracks for long-range transport of diverse cargos, have polar structures that define the directionality of transport by microtubule-based motors. Microtubule organization and polarity are critically important in cells with complex branches, where specialized cargo needs to be delivered to distal branches that can extend far from the soma (12-15). In the 1980s and 1990s, researchers assessed microtubule polarity using the “hooking” technique, which visualizes the orientation of assembling tubulin subunits via electron microscopy (16). More recently, live imaging of microtubule plus-end binding (EB) proteins and cryoelectron tomography (cryo-ET) have largely replaced hooking. In neurons, hooking (17), live-cell imaging in culture (18, 19), and *in vivo* imaging in rodents (18, 20) show that dendrites contain mixed-polarity microtubules (~65–70% plus-ends out), while axonal microtubules are almost uniformly plus-ends out, a finding confirmed by cryo-ET (21-23). Like axons, microtubules in oligodendrocyte processes are also oriented with plus-ends-out polarity (24, 25). Live imaging has also recently demonstrated that microtubule polarity in microglia, the brain’s resident immune cells, dynamically shifts with immune activation (26, 27). Though microtubule polarity is defined for neurons, oligodendrocytes, and microglia, it is yet to be defined for the structurally complex astrocyte.

In addition to microtubules, astrocytes also express actin and intermediate filaments (IFs), including glial fibrillary acidic protein (GFAP), which is commonly used as an astrocyte marker and often becomes upregulated in reactive states (9, 10, 28). However, few studies have explored the spatial organization and interplay between these three classes of the cytoskeleton in astrocytes. In neurons, extensive research highlights the importance of spatially organized cytoskeletal networks in establishing structural polarity and functional specialization across distinct cellular compartments. For instance, differences in microtubule polarity and post-translational modifications (PTMs) between axons and dendrites enable targeted transport of axonal versus dendritic cargo to support subcellular specification and function (29-31). In addition, distinct actin microstructures and patterning further support axonal and dendritic specialization as well as synaptic function (32-35), while neurofilaments, neuron-specific IFs, maintain axonal caliber (36-38). Thus, the importance of complex cytoskeletal regulation in neurons hints that the interplay between these three classes of cytoskeleton also underlies compartment-specific structure and function in astrocytes.

Unfortunately, fundamental questions about cytoskeletal organization in the branched processes of astrocytes have remained unresolved due to technical limitations. Resolving the fine detail of highly branched astrocyte processes *in vivo* via two-photon microscopy is technically challenging due to limitations in optical resolution and disentangling signal from noise in complex tissues. *In vitro* cell culture methods circumvent these issues and enable analysis of cell-autonomous mechanisms of cytoskeletal organization. However, traditional serum-based methods of culturing astrocytes produce flat, fibroblast-like cells with short protrusions and gene expression profiles resembling reactive astrocytes (39, 40). To address these limitations, we used immunopanning to isolate astrocytes from rodent brains. In immunopanning, a mixed population of cells is passed over a series of cell-surface antibodies to remove unwanted cell types and positively select a cell type of interest with high purity and cell viability. We cultured immunopanned astrocytes in serum-free, chemically defined conditions that preserve an *in vivo*-like branching architecture (39-41). To comprehensively visualize the astrocyte cytoskeleton in its native state, we leveraged immunostaining with confocal microscopy as well as cryo-ET to image cell structures with high resolution. Our work reveals distinct spatial organization of different elements of the cytoskeleton in the complex branches of astrocytes. The IF GFAP and microtubules define thicker main processes, while actin extends the boundaries of the cell through intricate microprocesses (e.g., filopodia, lamellipodia) at the cell periphery, and reticular webbing around the soma. Our observations uncover a spatially patterned cytoskeletal architecture that balances structural stability in proximal portions of branches with the capacity for functional specialization and dynamic remodeling at distal process tips.

## Results

### Astrocytes display a complex branching morphology that is unique from other glial cells

**Fig. 1.**
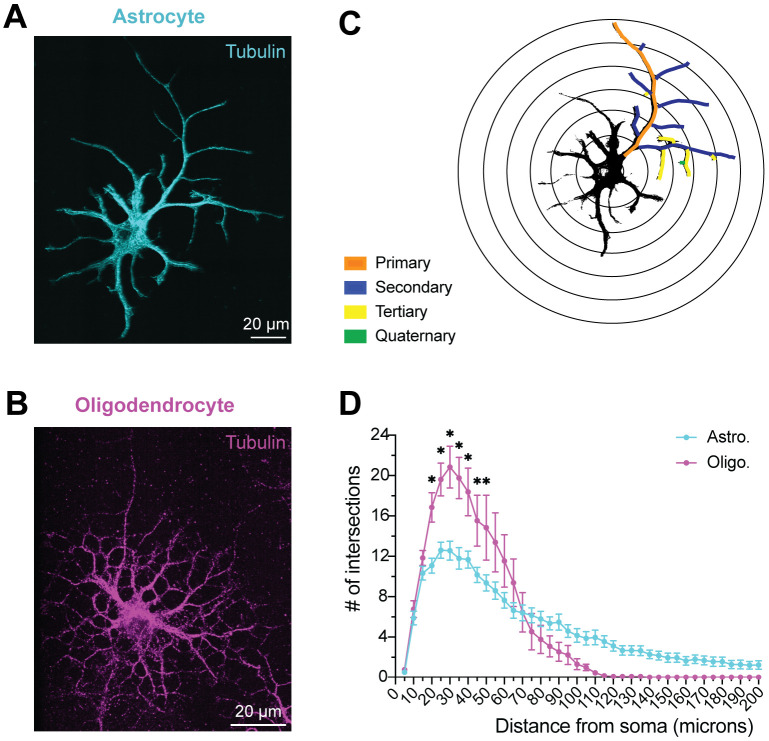
Immunopanned astrocytes exhibit a distinct, highly ramified microtubule architecture. **(A, B)** Representative immunofluorescence staining of tubulin (tyrosinated α-tubulin (YL1/2 antibody)) in immunopanned primary glial cells cultured in serum-free media. **(A)** DIV14 (days *in vitro*) astrocyte. **(B)** DIV3 differentiated oligodendrocyte. **(C)** Example image of Sholl analysis, showing tubulin staining of astrocyte in (A) overlaid on concentric rings surrounding the soma. Color coding illustrates branch order (primary, secondary, etc.) used throughout the study. **(D)** Quantification of Sholl analyses of astrocytes and oligodendrocytes. Astrocytes have significantly fewer branches than oligodendrocytes at distances between 20 μm and 50 μm away from the center of the soma. Mean ± SEM, n = 13–25 cells from 3–4 independent cultures per cell type (from 3–4 litters of rats). Mixed-effects model with repeated measures followed by Bonferroni multiple comparisons testing; * indicates p ≤ 0.05.

We purified primary astrocytes from neonatal rat brains using immunopanning and allowed the cells to mature in serum-free culture conditions. Following previous immunopanning protocols (39, 41), astrocytes were grown for two weeks to allow them to mature. This method yields astrocytes that are over >98% pure, with transcriptomes resembling healthy astrocytes *in vivo* (39, 40, 42). These immunopanned astrocytes are highly ramified, as visualized by α-tubulin staining (Fig. 1A). Immunopanned astrocytes have ~11 primary branches (Fig. 1B, fig. S1A), which is similar to previous estimates of ~5–10 primary branches in dye-injected astrocytes in rat brain slices (43).

We hypothesized that the branching pattern of microtubules in astrocyte processes is distinct from that observed in other ramified glial cell types. Since microglia are much smaller (44) and arise from a different lineage than other glia (45), we compared astrocytes to immunopanned oligodendrocytes that were matured for three days in differentiation media (Fig. 1B). We observed striking differences by Sholl analysis, which examines how branching complexity varies depending on distance from the soma or cell body (Fig. 1C). Oligodendrocytes have more proximal branches, peaking around 35 μm away from the cell body, and branch complexity drops off sharply after that, with few branches present >100 μm from the cell body. In contrast, astrocytes have significantly fewer proximal branches in the range of 20–50 μm away from the soma, their branching complexity declines more gradually, and branches are still present up to 200 μm away from the soma (Fig. 1D).

To further quantify branching complexity in astrocytes and oligodendrocytes, we also segregated processes by branch order (e.g., primary, secondary, etc.) and compared their frequency, proportion, and length. We categorized primary branches as the longest continuous branch extending from the cell body. Secondary branches sprout from the primary branches, tertiary from secondary, and so forth (Fig. 1C). We found both shared patterns and subtle differences between these two cell types. In both astrocytes and oligodendrocytes, secondary branches were the most frequent and abundant (fig. S1, A and B). Both cell types had similar branch order proportions (fig. S1B), and displayed a decrease in length with higher branch order (fig. S1C). While primary and secondary branch numbers were comparable, astrocytes have more tertiary and quaternary branches (fig. S1A). Conversely, higher-order branches are longer in oligodendrocytes (Fig. S1C). Together, these results show distinct tubulin branching patterns in immunopanned astrocytes and oligodendrocytes.

### Astrocyte microtubule polarity is primarily plus-ends out

**Fig. 2.**
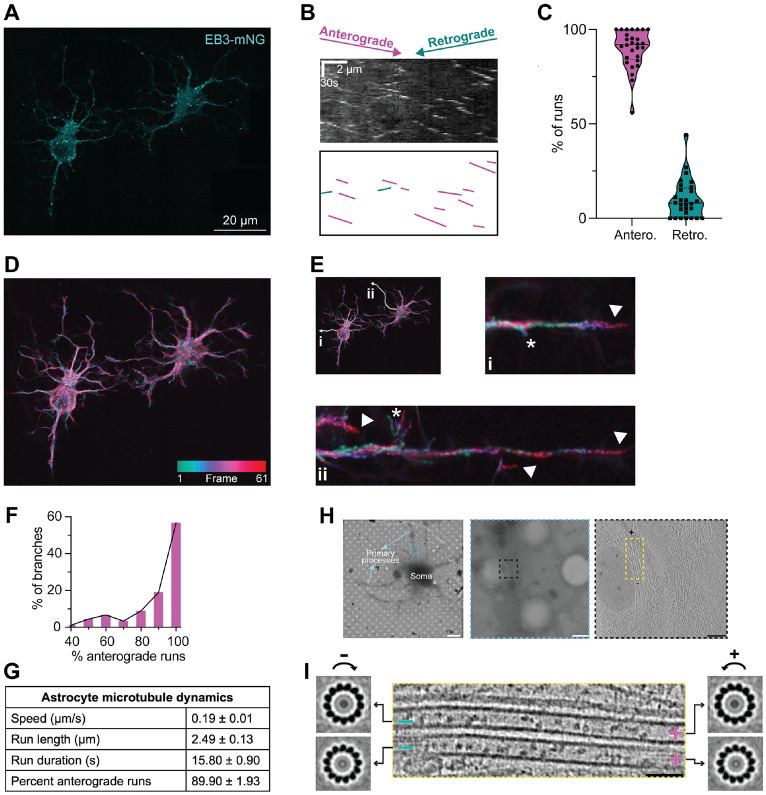
Astrocyte microtubules are predominantly oriented plus-ends-out. **(A**) Single frame from a live-cell movie of primary astrocytes expressing EB3-mNeonGreen (EB3-mNG) to mark the growing ends of microtubules. Processes from an adjacent astrocyte on the right were digitally erased for clarity. **(B)** Representative kymograph of one primary process from an EB3-mNG-expressing astrocyte, illustrating anterograde (purple) and retrograde (green) tracks. **(C)** Violin plot of the percentage of anterograde and retrograde EB3-mNG runs per cell. Violin plots are illustrated with dashed lines representing median and dotted lines representing interquartile range (IQR). **(D)** Heat map of a time-lapse of a movie of EB3-mNG-expressing astrocytes in (A). EB3-mNG runs are represented as transitioning from green (beginning of movie) to red (end of movie). Frame rate was 3 seconds per frame; movie length was 3 minutes. **(E)** Magnified images of digitally straightened branches in (D), highlighting EB3-mNG localization at the ends of growing branches. Branches and magnified insets are labeled i and ii. Arrowheads label distal branch tips, with neighboring branch tips visible in ii. Asterisks label potential nascent branch points. **(F)** Distribution of the percentage of anterograde and retrograde EB3-mNG runs per branch. **(G)** Table summarizing analysis of EB3-mNG runs in primary astrocytes, mean ± SEM (per cell). n = 110 kymographs from 28 cells. **(H)** Top left: example of an astrocyte grown on a cryo-EM grid. The he blue dashed box h ighlights the region of the primary process used for acquiring a tomographic tilt series, Scale bar: 5 μm. Top middle: zoomed-in image of the primary process, highlighting the region of the tomogram using a black dashed box. Scale bar: 500 nm. Top right: 2D slice of a reconstructed tomogram highlighting the microtubules in the yellow dashed box used for determining polarity and protofilament number. Scale bar: 100 nm. **(I)** A zoomed in view of the two adjacent microtubules (center), and end-on view of their corresponding subtomogram averages showing 13-protofilament architecture and plus-ends-out polarity, based on protofilament skew. Plus and minus ends are indicated. 93% of microtubules exhibited minus ends oriented toward the cell body and plus ends directed towards the tip of the process. n = 72 microtubules.

Microtubule polarity lays the groundwork for the directionality of motor-based cargo transport, which is particularly important in cells with long complex branches. Surprisingly, astrocyte microtubule polarity has not yet been defined. To address this gap, we expressed EB3-mNeonGreen in immunopanned astrocytes to label the growing plus ends of microtubules (Fig. 2A) and performed live-cell imaging (Movie S1). Kymograph analysis of EB3 runs showed that microtubule polarity in astrocytes was predominantly plus-ends out, with 90% anterograde runs per cell (Fig. 2, B and C). Time-lapse images of EB3 movement highlighted its localization at growing process tips (Fig. 2, D and E) and potential nascent branch points (Fig. 2E, asterisks).

We wondered whether all astrocyte branches were plus-ends-out or if there were some astrocyte branches with mixed polarity, similar to polarity differences in axons versus dendrites(18). Thus, rather than calculating averages per cell, we binned events for each branch. We observed that most branches (88%) had predominantly plus-ends-out polarity, with ≥70% anterograde runs. However, a small fraction of branches (12%) had more mixed polarity with 40–70% anterograde runs (Fig. 2F). In addition to polarity, we determined the average speed, run length, and run duration per cell of EB3 comets (Fig. 2G and fig. S2, A to C).

We further confirmed our polarity findings using cryo-ET (Fig. 2H), which allowed us to view microtubules in their native state without the expression of an exogenous tagged protein. Analysis of cross sections from subtomogram averages revealed the polarity and protofilament number of microtubules in the primary processes. The radial skew (clockwise versus counterclockwise) of microtubule cross sections (46) revealed that most microtubules (93%) were oriented with plus-ends-out polarity (Fig. 2I). In addition, we observed most microtubules in primary processes contain 13 protofilaments (Fig. 2I and fig. S2D), consistent with the canonical structure observed for neuronal microtubules (22, 47). The 13-protofilaments geometry and plus-ends-out polarity was maintained even in the presence of lattice breaks (fig. S2D). Overall, these data indicate that microtubules in primary processes are highly ordered, both in terms of protofilament structure and polarity, and suggest underlying mechanisms for maintaining their structure stability.

### Microtubules in astrocyte primary processes display markers of stabilization

**Fig. 3.**
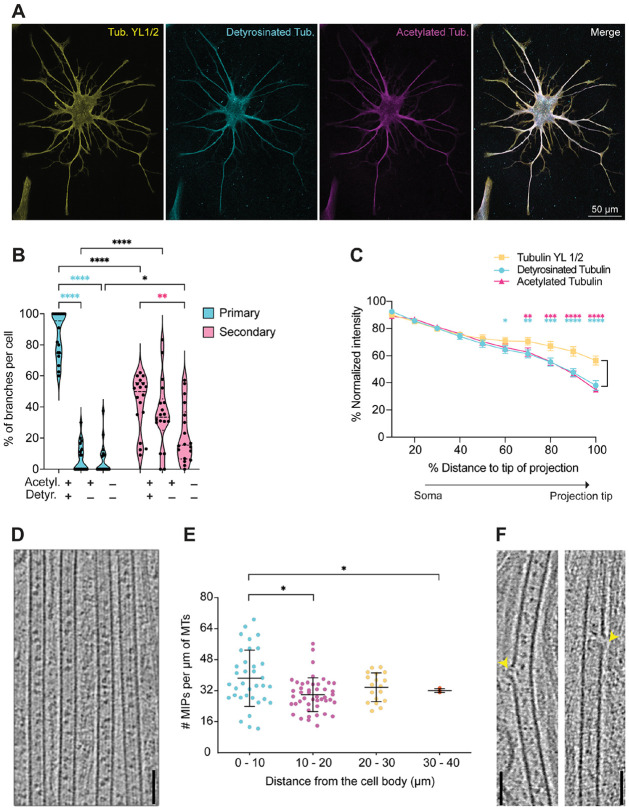
Microtubule-stabilizing PTMs and MIPs are present in the proximal regions of primary astrocyte branches. **(A)** Representative immunostaining for tyrosinated α-tubulin (YL1/2 antibody, yellow), detyrosinated tubulin (cyan) and acetylated tubulin (magenta) in a primary astrocyte. Merged image shows PTMs occur predominantly in primary branches with progressive loss of both PTMs toward distal tips. **(B)** Violin plots depicting percentages of primary and secondary branches positive for each PTM combination. Cyan asterisks represent comparisons within primary branches. Magenta asterisks represent comparisons within secondary branches. Black asterisks represent comparisons between primary and secondary branches. 2-way ANOVA with repeated measures followed by Tukey’s multiple-comparisons testing. Mean ± SEM of groups from left to right are 86.9 ± 3.6, 7.0 ± 2.2, 6.0 ± 2.4, 42.6 ± 4.2, 35.4 ± 5.3, and 22.1 ± 4.3%. **(C)** Fluorescence intensity of tyrosinated, detyrosinated, and acetylated tubulin along the length of primary branches. Both acetylated and detyrosinated microtubule intensities decrease significantly compared to tyrosinated microtubule intensities within the distal 30% of the branch. Fluorescence intensity was normalized against its maximum value per branch and distance is shown as a percentage of the total length of each branch (0% = edge of soma, 100% = projection tip). 2-way ANOVA with repeated measures followed by Dunnett’s multiple comparisons testing. Cyan asterisks show comparisons between tyrosinated tubulin and detyrosinated tubulin; magenta asterisks show comparisons between tyrosinated tubulin and acetylated tubulin. In (B) and (C), n = 16–18 cells from 3 independent cultures (from 3 litters of rats). * p ≤ 0.05, *** p ≤ 0.001, **** p ≤ 0.0001. **(D)** A 2D slice of a reconstructed tomogram showing the variable amounts of microtubule intraluminal proteins (MIPs) present in microtubules of astrocyte primary processes. Scale bar: 100 nm. **(E)** Graph showing MIP frequency per μm of microtubule length in astrocytic primary processes, binned by distance from the cell body: 0–10 μm (n = 35 microtubules), 10–20 μm (n = 46), 20–30 μm (n = 18), and 30–40 μm (n = 4). Bars represent mean ± SD. ** p < 0.01 by pairwise comparisons. **(F)** Representative examples of tomograms depicting microtubule lattice defects highlighted with yellow arrowheads. Scale bar: 100nm.

One mechanism that facilitates microtubule stability is tubulin PTMs, which can modulate microtubule dynamics and functions such as cargo transport (29, 48, 49). Thus, we analyzed tubulin detyrosination and acetylation (Fig. 3A), both of which have been associated with microtubule stabilization (50-54). Most primary branches in astrocytes were composed of microtubules exhibiting both acetylation and detyrosination (87%), with a significantly smaller percentage of primary branches displaying only acetylation (7%) or having neither modification (6%). In contrast, the proportion of secondary branches containing microtubules that were both acetylated and detyrosinated (43%) was only about half that of primary branches. The remainder of secondary branches contained microtubules that were either only acetylated (35%) or lacking either PTM (22%). Interestingly, we found no primary or secondary branches where detyrosination was present without acetylation (Fig. 3B). Thus, these results suggest that acetylation and detyrosination preferentially stabilize primary versus secondary branches in astrocytes.

We also examined whether levels of tubulin acetylation and detyrosination varied along the length of primary astrocyte branches. As astrocytes extend their processes, growing distal regions are likely newly formed and therefore more dynamic. Thus, we hypothesized that these PTMs are reduced at branch tips. In the proximal portion of primary branches (up to 50% of total branch length nearer to the soma), the relative fluorescence intensity of acetylated or detyrosinated tubulin was comparable to that of tyrosinated tubulin, which is detected by the α-tubulin (YL1/2) antibody (55, 56). However, in the distal portion of branches (70–100% of total branch length), both acetylated and detyrosinated tubulin intensities were significantly lower than tyrosinated tubulin (Fig. 3C). Thus, the decline in the level of these PTMs in the distal primary processes may indicate that microtubules are less stable near branch tips.

Other structural features known to enhance microtubule stability are the presence of intralumenal proteins (MIPs) and lattice defects (57-60). Using cryo-ET, we observed that nearly all microtubules in astrocytic primary processes contain MIPs (Fig. 3D). The distribution of MIPs ranges from 15–70 per μm of microtubule, with a modest enrichment of MIP frequency in the first 10 μm of primary processes (Fig. 3E). In addition to MIPs, we observed 1–4 defects per μm in the microtubule lattice (fig. S3A); these defects range from the absence of a single tubulin dimer, to lattice breaks involving multiple dimers in one or more protofilaments (Fig. 3F). In contrast, lattice defects are much more rare in neurons, with one defect per 20 μm (61). In all analyzed cases, the 13-protofilament geometry was maintained following a lattice defect (fig. S2D), which has been interpreted to correspond to microtubule rescue events (62). Though protofilament transitions from 12 to 13 have been reported in individual microtubules in *Drosophila* neurites, they were not observed in mammalian neuronal axons (22) . Thus, the high incidence of lattice defects in the absence of protofilament transitions suggests that astrocyte microtubules display robust stability.

The resolution of the tomograms also allowed us to visualize the morphology of individual microtubule ends, where distinctive morphologies have been associated with growth and stability versus depolymerization (63). We found that 18 out of the 34 microtubule ends analyzed displayed relatively short, flared morphologies, while the remaining 16 microtubule ends appeared blunt (fig. S3C). Blunt and short flared ends have been observed for *in vitro* GMPCPP-bound microtubules and thus are thought to correspond to stable microtubules (47). We did not observe ends with larger, curly protofilaments, which are associated with shrinking microtubules (64). The presence of MIPs and the prevalence of these microtubule end morphologies, both of which have been associated with microtubule stability (59, 60, 62), supports the idea that microtubules in the primary processes of astrocytes are highly stable.

### The cytoskeletal architecture of astrocytes is dominated by IFs

**Fig. 4.**
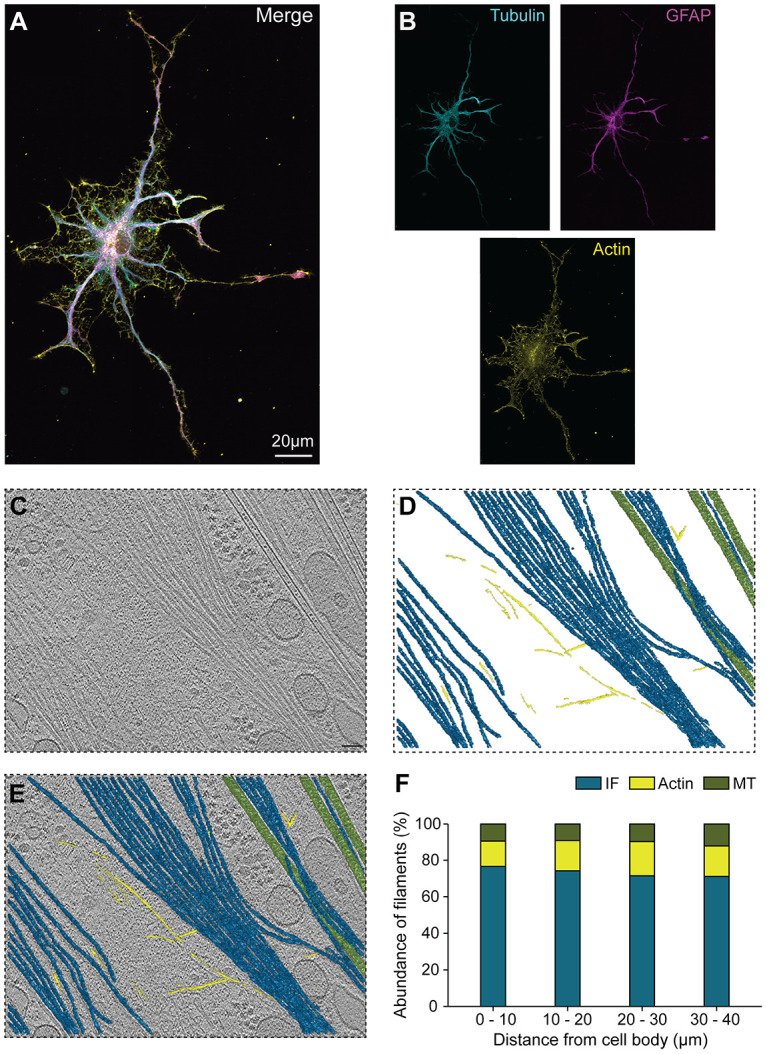
Abundant intermediate filaments define primary astrocyte processes while actin extends cellular boundaries. **(A-B)** Representative image of immunostaining for α-tubulin (YL1/2 antibody, cyan), the astrocyte-specific intermediate filament (IF) GFAP (magenta), and actin (phalloidin, yellow) in primary astrocyte. Processes from an adjacent astrocyte on the left were digitally erased for clarity. **(C)** 2D slice of a reconstructed and 3D CTF-corrected tomogram showing cytoskeletal filaments present in the primary process of an astrocyte. Scale bar: 50 nm. **(D)** Trained neural network-based segmentation (on Dragonfly) of cytoskeletal filaments, with microtubules in green, IFs in blue, and F-actin in yellow. **(E)** Overlay of the segmented cytoskeletal features in (D) on the tomogram in (C). **(F)** Graph showing percent abundance of filament classes in astrocyte primary processes, divided into 10-μm bins from the edge of the cell body to 40 μm away. At the different locations analyzed, IFs comprise ~70% of all filaments. The number of filaments represented in each bin are shown in fig. S4A.

Interactions between different classes of cytoskeletal filaments play key roles in cellular physiology. To investigate this crosstalk in astrocytes, we examined the organization of cytoskeletal elements across scales, from whole-cell architecture to the nanoscale. Immunofluorescence revealed substantial overlap between the astrocyte-specific IF GFAP and tubulin in thicker primary branches. Actin was present throughout the cell and extended cellular boundaries beyond the microtubule-IF framework (Fig. 4, A and B). Thus, the predominant method of using GFAP staining to visualize astrocytes misses a significant amount of the astrocyte cell space that contains actin.

To visualize the interplay between these cytoskeletal systems at higher resolution, we applied cryo-ET (Fig. 4C) and used segmentation of our cryo-tomograms to highlight individual filaments (Fig. 4D-E). Using our tomograms, we estimated filament length in astrocyte primary processes, finding IFs and microtubules averaged ~1.8 μm, whereas actin filaments were shorter, averaging ~0.8 μm (fig. S4, A and B). Notably, we observed that astrocytes in the proximal 40 μm of primary processes contain a striking abundance of IFs compared to microtubules or actin, with IFs making up ~70% of filaments (Fig. 4F, fig. S4C). In other words, IFs outnumbered microtubules ~7:1 and actin filaments ~2:1 (fig. S4D). The high ratio of IFs to microtubules in astrocyte primary processes resembles the distribution in medium to larger caliber myelinated axons, whereas microtubules dominate in dendrites (65-67).

The predominant IF expressed by astrocytes is GFAP, which forms both homopolymers and heteropolymers with another IF, vimentin (68-70). These heteropolymers can be visualized by immunostaining that shows high co-localization of GFAP and vimentin throughout the cell (fig. S4E). However, because the ultrastructure of GFAP has not yet been resolved, distinguishing GFAP from vimentin in tomograms was not possible. Therefore, we infer that the IFs seen by cryo-ET are a combination of GFAP homopolymers and GFAP-vimentin heteropolymers.

### The IF GFAP is enriched at astrocyte process tips

**Fig. 5.**
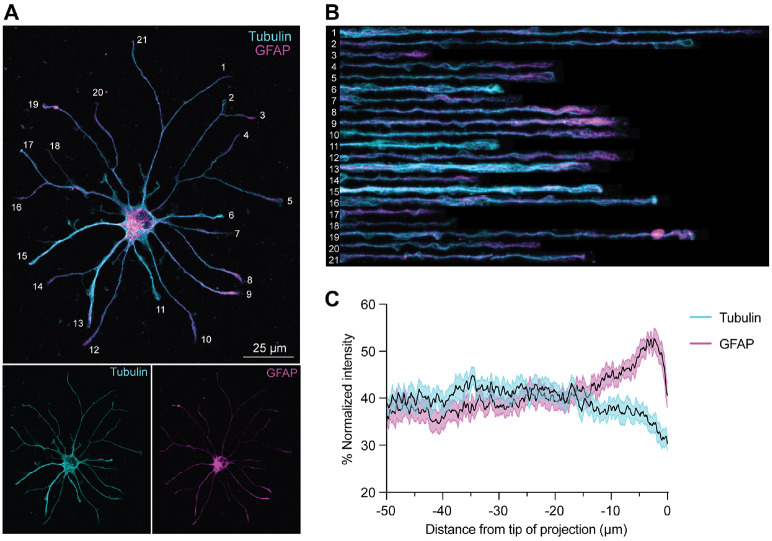
GFAP localization is enriched at distal process tips of primary astrocytes. **(A**) Representative image of immunostaining for α-tubulin (YL1/2 antibody, cyan) and GFAP (magenta) in a primary astrocyte. **(B)** Images of digitally straightened processes labeled in (A), showing co-localization of tubulin and GFAP and distal enrichment of GFAP. **(C)** Fluorescence intensity of tubulin and GFAP across the distal 50 μm of branch tips shows an increase in GFAP intensity and slight decline in tubulin intensity within the most distal 15 μm of process tips. Fluorescence was normalized to its maximum value across the entire branch. n = 25 cells from 3 independent cultures. 5–13 processes were analyzed per cell.

In neurons and other cell types, IFs interact with microtubules (71-73). In our astrocyte tomograms, microtubules were frequently positioned closely alongside IFs in primary processes (Fig. 4E and fig. S5A). To gain insight into the unexplored relationship between GFAP and microtubules in astrocytes, we examined their distribution in detail by immunofluorescence staining. Tubulin and GFAP signals largely colocalized along primary and secondary processes, but their intensities varied along the lengths of individual branches (Fig. 5, A to C). Notably, GFAP intensity was often more intense at process tips (e.g., Fig. 5B, Branches 8 and 9), though this was not universal across all branches (e.g., Fig. 5B, Branches 6 and 15). On average, GFAP intensity was significantly higher than tubulin intensity in the most distal 10 μm of processes (fig. S5B).

Quantification across the distal 50 μm of processes revealed relatively stable levels of both filaments until ~10–15 μm from the tip, where tubulin intensity declined and GFAP rose sharply (Fig. 5C). To better capture this shift, we calculated a distal enrichment ratio (average intensity across the most distal 10 μm divided by the average intensity over the preceding 40 μm). GFAP had a significantly greater distal enrichment ratio than tubulin (fig. S5C). Across individual branch tips, 34% displayed strong GFAP enrichment, while 28% showed no enrichment. In contrast, only 6% of branch tips exhibited strong distal tubulin enrichment, while most tips (72%) showed no enrichment (fig. S5D). Altogether, our results show that GFAP abundance frequently increases at the distal ends of tubulin-positive processes, suggesting a potential specialized role for GFAP at process tips.

### Diverse actin microstructures in astrocytes: lamellipodia, filopodia, and reticular webbing

**Fig. 6.**
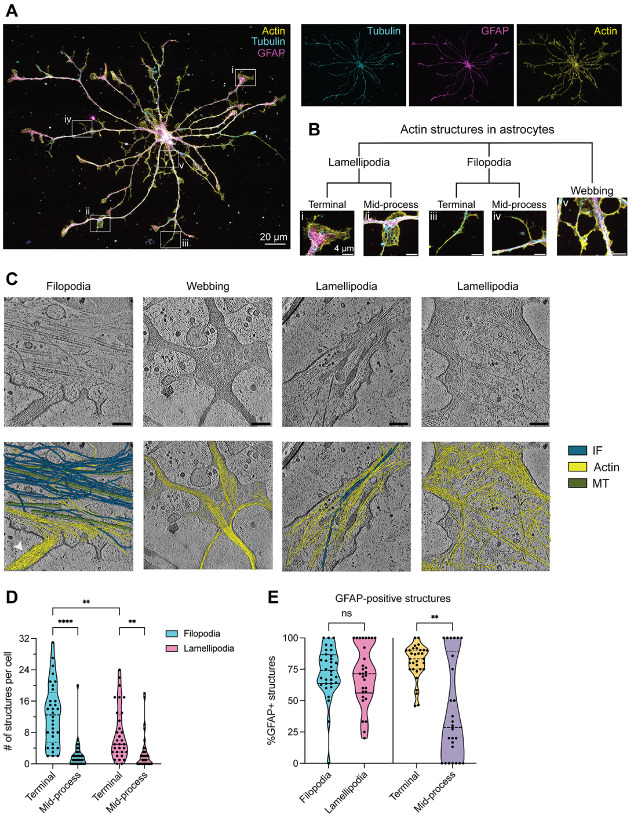
Diverse actin microstructures are present in the astrocyte periphery. **(A**) Representative image of immunostaining for α-tubulin (cyan), the intermediate filament GFAP (magenta), and actin (phalloidin, yellow) in a primary rat astrocyte. Boxed areas are magnified in and single channels are shown to the right. **(B)** Tree showing categorization of actin structures present in astrocytes, including terminal and mid-process filopodia and lamellipodia and webbing around the soma. Images are magnified from boxed areas of the cell in (A). All scale bars: 4 μm. **(C)** Top: example 2D tomogram slices of filopodia, lamellipodia, and webbing Bottom: overlay of segmentation of cytoskeletal filaments on the tomogram, with actin in yellow, microtubules in green, and IFs in blue. Scale bars: 200 nm. **(D)** Violin plots showing counts of filopodia and lamellipodia per cell, categorized by terminal versus mid-process location. On average, each astrocyte contained 12.6 ± 1.4 terminal filopodia, 2.2 ± 0.7 mid-process filopodia, 7.6 ± 1.2 terminal lamellipodia, and 2.8 ± 0.8 lamellipodia, mean ± SEM. 2-way ANOvA with repeated measures followed by Tukey’s multiple comparisons testing. **(E)** Violin plots showing the percentage of GFAP-positive filopodia versus lamellipodia (right) and terminal versus mid-process structures (left), Mann-Whitney tests. For (D and E), n = 33 cells from 4 independent cultures (from 4 litters of rats). ** p ≤ 0.01, *** p ≤ 0.001, **** p≤0.0001.

In addition to long, thick, shaft-like branches containing GFAP and microtubules, astrocytes have a fine meshwork of actin-rich microprocesses that significantly increases their surface area beyond the GFAP-positive skeleton (Fig. 4A and 6A). To visualize structures at the astrocyte periphery, we simultaneously stained for tubulin, GFAP, and actin, using phalloidin. These stains revealed a variety of actin microstructures and clear, consistent extension of actin localization beyond the main microtubule-IF framework. We classified actin structures as sheet-like lamellipodia, fine-tipped finger-like filopodia, or reticular webbing (Fig. 6, A and B) and also visualized them by cryo-ET (Fig. 6C).

Reticular webbing is an interesting and novel feature in astrocytes that are generally centered near the soma and links primary processes together (Fig. 6, A and B). Most cells (85%) exhibited webbing, which radiated maximally outward, on average, 49.5 ± 5.4 μm away from the edge of the soma. Astrocytic reticular webbing consists of aligned actin filaments and is often devoid of microtubules and IFs. In contrast, neuronal branchpoints visualized by cryo-ET have a mix of microtubules and unaligned actin (Mizuno 2022). Interestingly, small vesicles <100 nm in diameter are often found inside the reticular webbing.

We further quantified the relative distribution of filopodia and lamellipodia and their locations. On average, astrocytes had more filopodia (~15per cell) than lamellipodia (~10 per cell, fig. S6A). Lamellipodia and filopodia appeared both at process termini and along branches as mid-process protrusions (Fig. 6, A and B), but were more abundant at termini. A majority of filopodia and lamellipodia were terminal filopodia (52%) or terminal lamellipodia (32%, Fig. 6D). At mid-process sites, similar numbers and percentages of filopodia (8%) and lamellipodia (8%) were found (Fig. 6C and fig. S6B). Overall, mid-process filopodia and lamellipodia are rarer than terminal ones.

Since IFs can interact with actin and influence its dynamics (72), we hypothesized that GFAP may colocalize with actin structures. Indeed, the majority of filopodia (73%) and lamellipodia (71 %) were GFAP-positive (Fig. 6E). We asked whether the presence of GFAP correlates with distance from the soma. Indeed, GFAP localization correlated with location along the branch; terminal structures were almost twice as likely as mid-process structures to be GFAP-positive (81% versus 42%). We also quantified filopodia and lamellipodia number per cell, segregating GFAP positivity and location (fig. S6, C and D). We found that, among terminal structures, GFAP-positive ones were located significantly farther from the soma than GFAP-negative ones (68 μm versus 42 μm) (fig. S6E). Surprisingly, the magnitude of this difference (~26 μm) was much greater than the average difference (~10 μm) between terminal and mid-process structures (fig. S6F) Overall, our data indicate that the majority of filopodia and lamellipodia, as well as most terminal actin structures are GFAP-positive.

## Discussion

Our work provides compelling insights into the cytoskeletal architecture of complex astrocyte branches. Our results suggest a three-tiered arrangement of the cytoskeleton in astrocyte branches: (1) a stable inner core of acetylated and detyrosinated microtubules, further reinforced by IFs, (2) a transition zone where microtubules are less stable, and where enrichment of GFAP provides scaffolding to coordinate the microtubule and actin networks and support specialization of process tips, and (3) a morphologically diverse actin-based periphery that may be poised to respond to cues from neighboring cells and the environment. -Altogether, our findings suggest that coordinated regulation of the cytoskeleton underlies the structural and functional complexity of astrocyte branches.

### Cargo transport in astrocytes

Our work provides a robust system to study transport in ramified astrocytes and indicates that astrocytes harbor two mechanisms of microtubule organization that can enhance efficient cargo transport. First, astrocyte microtubules have a plus-ends-out orientation, with 89% anterograde EB3 runs and 93% of microtubules being plus-ends-out by cryo-ET (Fig. 2). This organization parallels the microtubule polarity observed in neuronal axons, suggesting a similar functional need for efficient anterograde and retrograde transport of diverse cargo toward process tips. Second, astrocytic microtubule PTMs may also play a role in supporting efficient transport via microtubule stabilization and facilitation of the action of certain motors. We found that most primary processes (87%) contain detyrosinated and acetylated microtubules (Fig. 2). In neuronal axons and cell lines, microtubule acetylation and detyrosination have been proposed to promote the selective binding to microtubules and motility of the anterograde motor kinesin-1 (74-78). Indeed, primary astrocytes are known to transport a variety of cargos, including autophagosomes (79), RNA granules (80), and specialized astrocyte proteins, such as gap junction proteins and the glutamate transporter GLAST1 (81). In these studies, many cargos moved in both anterograde and retrograde directions, but this work was only performed in non-ramified astrocytes. Our studies establish a framework for interpreting transport behavior in astrocyte branches, which we largely expect to be microtubule-based in primary processes. We hypothesize the largely plus-ends-out microtubule orientation helps support movement of diverse, specialized cargos toward distal branch tips, where astrocytes perform compartmentalized functions (e.g., water transport at interfaces with vasculature, or uptake of neurotransmitters near contact sites with synapses).

Intriguingly, the actin-rich reticular webbing centered around the soma that connects primary processes in astrocytes (Fig. 6) may also play a role in transport. By cryo-ET, this reticular webbing is often devoid of microtubules and IFs, but contains small vesicular organelles (Fig. 6C). Consistent with this, membrane-bound organelles have also been found *in vivo* in very fine processes of astrocytes near synapses (82, 83). Thus, these observations raise the intriguing possibility that myosin-mediated vesicular transport may occur along the actin filaments within the webbing.

### Stability of primary astrocyte processes

Astrocytes have highly stable primary processes and appear to utilize mechanisms affecting two classes of cytoskeleton to achieve structural stability. First, astrocyte microtubules contain PTMs like acetylation and detyrosination that are associated with microtubule stability (50-54). Consistent with this proposal, cryo-ET shows that astrocyte microtubules contain MIPs, which also correlate with highly stable microtubules in neurons (59). Second, astrocyte processes contain abundant GFAP. Indeed, GFAP and tubulin colocalize along the lengths of most primary processes (Fig. 4), which is consistent with an early study in non-ramified primary rat astrocytes that found co-localization of GFAP with acetylated microtubules (84). In addition, we observe a very high IF:microtubule ratio of ~7:1 by cryo-ET (Fig. 4), which is in a similar range as the ~10:1 neurofilament:microtubule ratio in myelinated portions of medium to larger caliber sensory and motor axons (axonal diameter >3 μm) (65-67). Since neurofilaments increase the viscoelastic properties of axons (85, 86), astrocyte IFs likely also play similar biophysical roles, maintaining structural integrity of longer primary processes in response to compressive and tensile stress. Interestingly, early work shows that GFAP knockout mice are far less resilient to blunt mechanical trauma, supporting a role for GFAP in structural stability (87). These two mechanisms, the presence of stabilizing tubulin PTMs and the abundance of IFs, likely work in synergy, as detyrosinated microtubules have been shown to increase their binding to IFs in other cells, enhancing their stability (73, 88, 89). Thus, both microtubules and IFs contribute to the structural stability of astrocyte primary processes.

In addition, the spatial organization of microtubule PTMs we observed may contribute to two important properties of *in vivo* astrocyte morphogenesis: dynamicity and tiling. Two-photon microscopy of astrocytes *in vivo* found that microtubules were mostly stable over 20-minute increments, but events of retraction, extension, and branching occurred at distal ends(90). These findings are consistent with our results showing that microtubule-stabilizing PTMs are not present at distal tips, with decreased abundance of acetylated and detyrosinated tubulin at the most distal third of primary processes (Fig. 3C). This spatial patterning would decrease microtubule stability at distal ends of processes, rendering them more permissive for local dynamic remodeling. Tiling is the process by which individual astrocytes establish and maintain distinct non-overlapping spatial domains within the brain (43, 91). As an astrocyte defines its territory from that of a neighboring astrocyte, branches may need to turn or change directions. Microtubule acetylation imparts increased flexibility to microtubules, buffering against mechanical stress by allowing bending (53, 54, 92). Indeed, in fibroblasts, microtubule acetylation has been linked to contact inhibition (93), a crucial cellular mechanism to prevent cells from growing into one another. Thus, microtubule acetylation may also play an important role in astrocyte tiling by supporting mechanical flexibility of microtubules as branches find distinct territories to occupy.

Finally, our quantification of microtubule PTMs suggests a temporal sequence of PTM acquisition. To our surprise, we observed no astrocyte branches that were detyrosinated but not acetylated (Fig. 3). These results suggest that acetylation precedes detyrosination during astrocyte maturation. Similarly, fibroblasts observed using super-resolution microscopy also did not harbor any non-acetylated-detyrosinated microtubules (94). It is unlikely that these results in astrocytes and fibroblasts are due to antibodies competing for nearby binding sites, because acetylation occurs inside the microtubule lumen while detyrosination occurs on the microtubule surface. However, PTM kinetics are also likely involved, because acetylation of microtubules can occur rapidly on the timescale of minutes while detyrosination accumulates more slowly (95). Thus, acetylation likely precedes detyrosination in astrocytes and this observation may extend to other cells as well.

### Subcellular structures for specialized astrocytic functions

*In vivo*, astrocytes produce 2 types of processes that are morphologically and molecularly distinct (96-100). These include perisynaptic astrocyte processes (PAPs) that closely associate with neuronal synapses, buffer glutamate, and dynamically remodel in response to neuronal activity (7, 101). Endfeet are critical for maintaining the blood–brain barrier and coupling blood flow to neuronal activity (3). The actin-rich structures that we observed in our cultures are consistent with features of PAPs and endfeet.

The filopodia that we observed in our astrocyte monocultures may parallel precursor structures to PAPs, due to three shared properties. First, terminal filopodia are very abundant in cultured astrocytes (Fig. 6D), as are PAPs, with an *in vivo* estimate of thousands per rodent astrocyte (43, 103). Second, previous 3D reconstruction of astrocytes by FIB-SEM and SBF-SEM showed synaptic contacts preferentially occur at fine terminal branches (“leaflets”) and sites along astrocyte processes that constrict (83, 100, 104). Similarly, filopodia in our cultured astrocytes are small, finger-like projections. Third, recent work demonstrates actin remodeling in astrocytes is crucial for structural plasticity of PAPs in response to neuronal activity (101). This parallels the role of axonal dendritic filopodia during neuronal development, which rapidly and repeatedly extend and retract in search of synaptic partners (105). A similar need for the precise sensing mechanisms and dynamic remodeling of filopodia likely operates at PAPs. Together, these observations suggest that filopodia may be well suited to give rise to PAPs, based on their abundance, morphology, and an *in vivo* role for rapid actin remodeling.

In contrast to PAPs, endfeet are broad and sheet-like in order to fully cover the vasculature and therefore are more consistent with the lamellipodia and GFAP-rich terminals that we have identified. Notably, in human and rodent brains, GFAP is enriched in astrocyte endfeet (103). Indeed, GFAP is distally enriched in cultured astrocyte processes (Fig. 5) and a subpopulation of terminal actin structures colocalize with GFAP (Fig. 6). The large surface area of endfeet should enable more extensive coverage of the vasculature and a more stable adhesive surface to withstand repeated changes in vessel mechanical pressure. Because IFs can regulate focal adhesion dynamics and actomyosin contractility (72, 106), distally enriched GFAP may tailor local actin responses to dynamic adhesive and mechanical forces, such as variable stiffness of the vasculature in response to vessel constriction and dilation. Thus, the interplay between GFAP and actin in distal astrocyte lamellipodia is especially poised to respond to vascular mechanical function. Future co-culture studies pairing astrocytes with neurons or vascular cells could assess whether actin-based filopodia and GFAP-rich lamellipodia contribute to the formation of PAPs and endfeet.

### Heterogeneity of Astrocyte Processes

A recent landmark study made the surprising observation that each astrocyte contacts at least one blood vessel. By two-photon microscopy, all of the ~700 astrocytes analyzed in the mouse brain cortex contacted vasculature, with most astrocytes contacting 2–5 blood vessels (102). This finding has important implications for astrocyte biology, as the proportion of astrocytes contacting a vessel was previously unknown and assumed to be only a smaller subset of cells. In other words, each astrocyte possesses heterogeneous branches – those that terminate in PAPs associated with neuronal synapses and those that terminate in endfeet associated with blood vessels. This is analogous to the compartmentalization of neuronal processes, where axons and dendrites adopt distinct morphologies and functions. One key difference between neuronal processes is microtubule polarity; while axons have uniform plus-ends-out polarity, dendrites have mixed polarity. In cultured neurons, axon specification is a process whereby a single neurite from an initial pool of sprouted neurites elongates into the axon, while other neurites mature into dendrites. In astrocytes, we observed a small percentage of processes (13.3%) that display mixed microtubule polarity (40–70% plus-ends out) (Fig. 2F). It is intriguing to speculate whether this subpopulation of mixed-polarity astrocyte processes might be functionally distinct, poised to give rise to either PAPs or endfeet. Alternatively, signals from neurons and the vasculature may be required for full maturation of PAPs and endfeet. Thus, future experiments co-culturing with neurons or vasculature are needed to elucidate whether the specification of heterogeneous processes in astrocytes is cell autonomous.

In conclusion, our findings lay the groundwork for further investigation into how cytoskeletal architecture and dynamics underpin astrocyte morphology and function. As a cellular system to investigate mechanisms of cytoskeletal organization, astrocytes present many fascinating and novel cell biology questions, including the complex interactions between multiple classes of cytoskeleton, the actin-rich reticular webbing that connects primary processes, and the ontological origin of process heterogeneity. Many concepts in neuronal cell biology, including cargo transport and local translation, likely play unexplored roles in establishing subcellular specificity and function in astrocyte processes (80, 107, 108). Moreover, these questions in astrocyte cell biology will have important implications in our understanding of the roles astrocytes play in many neurological diseases and open the door to exciting new topics of investigation.

## Methods

### Animals

All experiments were conducted using Sprague Dawley CD/SD rats (Charles River Laboratories, Strain Code: 001). Litters were sacrificed between P5–P7 (postnatal day) by rapid decapitation. Cortices used for cultures were combined by litter, generating cultures of mixed sex. Rats were housed on a 12-hour light/dark cycle with food and water *ad libitum*. All animal procedures were performed in accordance with protocols approved by either the University of California, Berkeley (AUP-2023-06-16512) or the National Institute of Neurological Disorders and Stroke Animal Care and Use Committee.

### Astrocyte immunopanning and culture

Methods for isolating and culturing immunopanned neonatal rat astrocytes were performed according to previous protocols (39, 41) with minor modifications.

The day before immunopanning, 15 cm Petri dishes were coated by adding 60 μL secondary antibody, or 100 μg BSL-1 lectin (Vector Laboratories, L-1100-5), to 20 mL of 50 mM Tris-HCl (pH 9.5), ensuring the entire dish surface was covered, and incubated overnight at 4 °C. Secondary antibodies used were goat anti-mouse IgG + IgM, goat anti-mouse IgM μ-chain specific, and goat anti-rat IgG (Jackson ImmunoResearch, 115-005-044, 115-005-020, 112-005-167). The following day, the secondary-antibody solution was discarded and plates were washed 3 times with PBS. 20 μL of each primary antibody was added to 12 mL of a DPBS solution with 0.2% BSA (Sigma, A4161) and DNase (Worthington, LS002007); this solution was incubated on the same plates at room temperature until needed for immunopanning steps.

Following preparation of antibody-coated plates, cortices from 8 P5–P7 rats with meninges removed were minced into ~1 mm^3^ pieces, split evenly between two 6-cm petri dishes, and digested for 45 minutes at 34 °C in 20 mL of enzymatic solution containing 0.0040 g l-cysteine (Sigma, C7477), 100 U (unit) papain (Worthington, LS003126), and 100 μL of 0.4% DNase. The papain solution was pre-equilibrated with carbogen (5% CO_2_, 95% O_2_) and carbogen was gently bubbled over cortices during digestion. The tissue was then washed 3X and triturated in low-ovomucoid solution to create a single cell suspension. Low-ovomucoid solution consisted of a pre-equilibrated buffer solution (0.46% D(+)-glucose and 26 mM NaHCO_3_ in Earl’s Balanced Salt Solution) containing 8.5 μM BSA (Sigma, A8806), 20 μM ovomucoid-based trypsin inhibitor (Worthington, LK003182), and 0.45 μM insulin (Sigma, I6634). Pre-equilibrated high-ovomucoid solution (21.2 μM BSA, 50 μM trypsin inhibitor) was carefully layered under the cell suspension in low-ovomucoid solution and the cells were spun down for 5 minutes at 120*g* at room temperature to fully quench papain activity. The cell pellet was then resuspended in panning buffer (0.02% BSA/DNAse and 0.45 μM insulin in DPBS) and filtered through a 40 μm cell strainer to remove clumps of cells and to generate a single-cell suspension. From this mixture, macrophages, microglia, endothelial cells, and oligodendrocyte precursor cells were removed by immunopanning with anti-mouse IgG+IgM secondary antibody, BSL-1 lectin, anti-CD45 antibody (BD Biosciences Pharmingen, 550539), and O4 hybridoma supernatant (homemade), respectively. Lastly, astrocytes were positively selected for using anti-ITGB5 antibody (eBioscience, 14-0497-82). Resulting astrocytes were lifted with 400 U trypsin (Sigma, T9935) in EBSS pre-equilibrated at 37 °C with 10% CO_2_ in the cell culture incubator. Astrocytes were then spun down in 30% heat-inactivated FBS (Gibco, 16000044) for 5 minutes at 130*g* at room temperature to quench trypsin activity. This was the only time during which cells were exposed to serum.

Astrocytes were then resuspended in growth medium and plated onto 12-mm sonicated glass coverslips pre-treated with 10 μg/mL poly-D-lysine (PDL) hydobromide (Sigma, P6407) in a 24-well cell culture plate, at a density of 20,000–40,000 cells/well. Cells were grown at 37 °C in 10% CO_2_ for 13–14 days in astrocyte growth medium containing 1:1 DMEM:Neurobasal mixture (Gibco, 11960-051, 21103-049) containing 100 U/mL penicillin/streptomycin (Gibco, 15140-122), 1 mM sodium pyruvate (Gibco, 11360070), 1X GlutaMAX (Gibco, 35050061), 1X DMEM-based SATO supplement (100 μg/mL BSA (Sigma, A4161), 100 μg/mL transferrin (Sigma, T1147), 16 μg/mL putrescine dihydrochloride (Sigma, P5780), 60 ng/mL progesterone (Sigma, P8783), and 40 ng/mL sodium selenite (Sigma, S5261)), 5 ug/mL N-acetyl-L-cysteine (Sigma, A9165), and 5 ng/mL HbEGF (Sigma, E4643). This media was refreshed every 4–7 days. In experiments investigating perisynaptic and perivascular markers, the growth medium also contained 50 ng/mL BMP4 (STEMCELL Technologies, 78211) and 10 ng/mL bFGF (STEMCELL Technologies, 78003) to further promote cell health and branch formation (109).

### Oligodendrocyte immunopanning and culture

Oligodendrocyte precursor cells (OPCs) were purified from neonatal Sprague-Dawley rat pups (P6–P8) (Charles River) of both genders by immunopanning (110). Briefly, cortical tissue was dissociated by papain digestion and filtered through a 40-μm cell strainer to obtain a single-cell suspension. This suspension was incubated in two negative-selection plates, one coated with anti-RAN-2 hybridoma supernatant and the other coated with anti-GC (glucocerebrosidase) hybridoma supernatant, then in a positive-selection plate coated with anti-O4 hybridoma supernatant. All hybridoma supernatants were made in-house. Adherent cells were trypsinized and plated on 12-mm glass coverslips that were cleaned via sonication for 1 hour in ethanol, then coated with 10 μg/mL poly-D-lysine (PDL) hydrobromide at a density of 10,000–20,000 cells each. Cells were cultured at 37 °C and 10% CO_2_ in proliferation media containing PDGF (STEMCELL Technologies, 78095.1) and NT3 (STEMCELL Technologies, 78074), or differentiation media containing NT3, CNTF (STEMCELL Technologies, 78010.1), and T3 (Sigma, T6397). Immunopanned oligodendrocytes are >99% pure and express markers of myelinating oligodendrocytes following differentiation (17065439, 25186741, 23906908).

### Immunofluorescence staining of cultured primary glial cells.

In experiments involving tubulin antibodies, fixation and staining was performed using PHEM buffer (60 mM PIPES, 25 mM HEPES, 10 mM EGTA, 2 mM MgSO_4_, pH 6.9–7) to better preserve microtubule architecture. Staining that did not involve tubulin used PBS-based buffers. Cultures were washed with 37 °C PHEM or PBS, and then fixed at 37 °C for 10 minutes with either fresh 4% PFA (Electron Microscopy Sciences, 15711) in PHEM containing 4% sucrose, or fresh 4% PFA in PBS. Then, coverslips were washed twice with PHEM or PBS, and permeabilized with 0.1% Triton X-100 in PHEM or PBS for 3 minutes. Following permeabilization, coverslips were washed 3X with PHEM or PBS, and then incubated in PBS-based (5% donkey serum (Sigma, D9663), 1% BSA (Sigma, A8806) in PBS) or PHEM-based blocking buffer (5% donkey serum, 1% BSA, 0.3 M glycine in PHEM) for 60 minutes at room temperature. Coverslips were incubated with primary antibodies diluted in blocking buffer overnight at 4 °C. The following day, coverslips were washed 3X with PHEM or PBS, then incubated with secondary antibodies and/or Phalloidin-647 diluted in blocking buffer for 1 hour at room temperature with protection from light. Then, coverslips were washed 3X with PHEM or PBS and mounted to slides using non-hardening Vectashield Plus (Vector Laboratories, H-1900). Slides were imaged on the same day or the following day using 60X or 100X objectives on a spinning-disk confocal (Nikon Eclipse-T2 inverted microscope with Yokogawa X1 spinning disk) with a sCMOS camera (Hamamatsu Orca-FusionBT).

#### Primary antibodies and dyes (with dilutions):

Tyrosinated a-Tubulin YL1/2 (ThermoFisher, MA1-80017, 1:500)

Detyrosinated Tubulin (Abcam, ab48389, 1:200)

Acetylated Tubulin (Sigma, T7451, 1:200)

GFAP (Cell Signaling, 12389S, 1:400) (Invitrogen, 13-0300, 1:200)

Phalloidin-647 (Sigma, A30107, 1:400)

Vimentin (Abam, ab24525, 1:400)

#### Secondary antibodies (with dilutions):

DyLight^™^ 405 AffiniPure^®^ Donkey Anti-Rat IgG (H+L) (Jackson ImmunoResearch, 712-475-153, 1:250)

Alexa Fluor^®^ 488 AffiniPure^®^ Donkey Anti-Mouse IgG (H+L) (Jackson ImmunoResearch, 715-545-151, 1:500)

Alexa Fluor^®^ 488 AffiniPure^®^ Donkey Anti-Rabbit IgG (H+L) (Jackson ImmunoResearch, 711-545-152, 1:500)

Rhodamine Red^™^-X (RRX) AffiniPure^®^ Donkey Anti-Mouse IgG (H+L) (Jackson ImmunoResearch, 715-295-151, 1:500)

Rhodamine Red^™^-X (RRX) AffiniPure^®^ Donkey Anti-Rabbit IgG (H+L) (Jackson ImmunoResearch, 1:500)

Rhodamine Red^™^-X (RRX) AffiniPure^®^ Donkey Anti-Rat IgG (H+L) (Jackson ImmunoResearch, 712-295-153, 1:500)

Alexa Fluor^®^ 647 AffiniPure^®^ Donkey Anti-Rabbit IgG (H+L) (Jackson ImmunoResearch, 711-605-152, 1:500)

### Live-cell imaging

Immunopanned astrocytes used for live imaging were cultured on 35-mm glass-bottom dishes (Mattek, P35G-1.5-10-C) coated with PDL hydrobromide. Cells were seeded at a density of ~60,000 cells per dish, transfected with EB3-mNeonGreen on DIV9 - 10, and imaged on DIV13 - 14. Transfection was performed with Lipofectamine 3000 (Invitrogen, L3000-001) according to manufacturer instructions, using 0.5 μg of plasmid per dish. EB3-mNeonGreen was a gift from Dorus Gadella (Addgene plasmid #98881; http://n2t.net/addgene:98881; RRID:Addgene_98881).

For live-cell imaging, the DMEM:Neurobasal base of astrocyte growth media was replaced with FluoroBrite DMEM (Gibco, A1896701) to limit background fluorescence. The cells were imaged in an environmental chamber at 37 °C with 5% CO2, and imaging was limited to one hour or less per plate to limit phototoxicity. Images were collected every 3 s for 3 min on a spinning-disk confocal (Nikon Eclipse-T2 inverted microscope with Yokogawa X1 spinning disk) with a sCMOS camera (Hamamatsu Orca-FusionBT).

### Immunofluorescent Image analysis

All image quantification was performed using the Fiji image analysis software (Ver. 2.16.0; Build 26d66057dd). Fluorescent intensities of tubulin PTMs, GFAP, and phalloidin were quantified by tracing each process with the Segmented Line Selection tool (20-pixel width), then exporting the data from the Plot Profile tool. The relative intensities of different stains were compared against one another by expressing fluorescent intensity values along individual processes as a percentage of the maximum intensity value of each marker along the process. These values are denoted as “% normalized intensity” in figures. Linearized representations of processes were generated by using the Straighten tool. For quantification and representation of tubulin PTMs as a percentage of total process length, mean values were binned into deciles.

### Sholl analysis

Images of tubulin-stained oligodendrocytes and astrocytes were traced using the Adobe Photoshop brush tool with a 5-pixel round tip for conversion into binary images. The freehand tool in Fiji was used to trace the cell body and to calculate a center of mass, which was used subsequently as the coordinates for the center of the cell. These coordinates and binary images were then input into the Sholl Analysis plugin (SNT 4.2.1) in Fiji and analyzed with a radius step size of 5 μm.

### Quantification of process number and length

The lengths of astrocyte microtubules were calculated by tracing tubulin-immunostained processes with the segmented line tool in Fiji. Primary processes were defined as a process extending directly from the edge of the cell body. Length was defined as the longest continuous path along the primary process and measured from the distance between the cell body edge and the process tip. Processes branching off from a primary process were defined as secondary processes. Tertiary processes were defined as those branching off from secondary processes, while quaternary processes were defined as those extending off of tertiary processes. Secondary, tertiary, and quaternary processes were traced from the base of the initial branch point out to the distal process tip. In cases where multiple continuous paths could be traced, the longest continuous path was assigned to the lowest order branch.

### Kymograph generation and microtubule polarity analysis

Kymographs were generated from live-cell images of EB3-mNeonGreen-transfected astrocytes using the Multi Kymograph tool in Fiji. Up to four kymographs from each cell were analyzed. The length and angle of each EB3 comet was measured using the Segmented Line tool and used to calculate direction, velocity and net displacement of each run. Two to four kymographs from each cell were analyzed. Kymographs were taken from predominantly primary processes with a smaller percentage (~15%) taken from secondary processes. Average velocity, displacement, and percentage of distally- and proximally-traveling runs were calculated for each cell. Temporal heat maps of EB3 displacement were generated using the Temporal-Color Code tool in Fiji.

### Actin microstructure classification and quantification

Actin microstructures on tubulin-, actin-, and GFAP-immunostained astrocyte processes were classified and counted in Fiji based on the presence (filopodia) or absence (lamellipodia) of thin, pointed actin shapes protruding from the tip of the microstructure. We also quantified the location of filopodia and lamellipodia along processes (terminal or mid-process) and their distance from the cell body. Filopodia or lamellipodia microstructures localized to the distal end of a process were defined as terminal, while microstructures protruding from any other location along the length of the process were defined as mid-process. Distance from the cell body was measured using the Segmented Line tool in Fiji and defined as the shortest continuous path from the edge of the cell body to the most distal point of actin localization at the tips of filopodia, or most distal portion of actin protrusion for lamellipodia.

Filopodia and lamellipodia microstructures were further categorized by the presence or absence of GFAP within the microstructure. GFAP-positivity was defined by GFAP localization that extends to the actin-positive edge of the structure. Webbing-like microstructures were defined as filaments connecting two processes together and forming an enclosed extracellular space. For these, the distance from the cell body was defined using the Straight Line tool in Fiji from the edge of the cell body to the most distal webbing-like microstructure.

### Sample preparation for cryo-ET

R2/2 200 mesh quantifoil gold grids (Electron Microscopy Sciences) were cleaned with chloroform, placed in a sterile 35-mm glass-bottom dish (MatTek Life Sciences), glow-discharged using a Tergeo-EM plasma cleaner (PIE Scientific), and coated with poly-D-Lysine overnight. The dishes were washed three times with water before plating the astrocytes (20000 cells/dish) isolated using the procedure described above. The plated cells were incubated on the grid at 37 °C with 10% CO_2_ for 13 days as shown in Fig 2H. For cryo-ET study, the grid was blotted for 10 s with a blot force of 10 in a 95% humidity chamber of a Vitrobot Mark IV (Thermo Fisher Scientific) and vitrified by plunging into liquid ethane.

### Cryo-ET data acquisition

Data was acquired at the Cal-Cryo Facility at UC Berkeley using a Titan Krios G3i with a BIO Quantum energy filter (Gatan) operated at 300 kV (Thermo Fisher Scientific), using a K3 direct electron detector (Gatan). Tilt series were collected using Serial EM software (111) at a magnification of 33,000x (pixel size of 2.63 Å), between −60° to + 60° tilt angles with 3° increments, an electron dose of 2.42 e^−^/Å^2^ per image (total dose of 99.62 e^−^/Å^2^), and at defocus values ranging between −3 to −6 μm.

### Cryo-ET data preprocessing and tomographic reconstruction

131 tilt series composed of 41 images each were acquired by manually selecting regions on primary processes of the astrocytes (as shown in Figure 4B and 4C). Beam-induced motion correction was performed on raw tilt images using Relion5 implementation of the UCSF MotionCor2 program (112, 113), and the x8 binned images were then aligned for 3D reconstructing using automatic alignment in the Eman2 software, followed by CTF (Contrast Transfer Function) estimation to screen for good quality tomograms (114). Fifty-five selected tomograms were reconstructed at bin x4 (10.52 Å/pixel) using the Batch Tomogram Reconstruction, with Batchruntomo, and CTF-corrected using the Ctfplotter on IMOD (version 5.1)(115, 116). The reconstructed bin x4 tomograms were CTF deconvolved and corrected with IsoNet to enhance resolution and CTF correction, which help determine the microtubule polarity based on the protofilament skew (117).

### Tomogram segmentation

The cytoskeletal components of astrocytes in our tomograms were segmented using the non-commercial version of Dragonfly 2024.1 (Object Research Systems) following the protocol described in Heebner et al., 2022 (118). Briefly, a gaussian filter was applied to the x4 binned tomogram to reduce noise and enhance signal. A small subregion containing cytoskeletal features was selected for manual annotation of about 7-10 tomographic slices. The segmentation wizard was then used to carry out iterative training of a 2.5-D U-Net segmentation model with 5 slices, initial filter count of 64, and depth level 5, until a good training model was obtained. This model was then used to predict the cytoskeletal features in whole tomograms. The predicted segmentations were manually cleaned up to eliminate segmented noise.

### Subtomogram averaging of microtubules

Microtubules from 18 different primary processes across 9 cells were used for averaging. From 31 x4 binned tomograms of primary processes, individual microtubules were manually traced by placing a marker at the approximate center using the *Volume Tracer* tool, and 3D particle picking was performed using the *Pick Particle* tool in Chimera (version 1.19) (119). Particle extraction and averaging was performed using subTOM (version 1.1.6) (https://github.com/DustinMorado/subTOM). The particles were extracted with a box size of 64 pixels centered on the coordinates picked on Chimera. Iterative alignment of the extracted subtomograms was carried out, followed by averaging. Z-slices of these averages were used to determine the microtubule protofilament number and the skew direction of the tubulin subunits that defines the polarity of the microtubule (46, 120). Of the 81 microtubule averages, 8 were inconclusive concerning protofilament number and/or polarity. The microtubule particle picking direction is key to determine the polarity: when the particle picking was from minus to the plus end, the averages show minus end polarity (clockwise direction). The protofilament skews of individual microtubules observed on the z-slices of the deconvolved tomograms therefore defined the microtubule polarity relative to the cellular compartment.

### Determination of MIP and lattice defect frequencies of individual microtubules

Microtubules from 12 different primary processes across 7 cells were used to determine MIP and lattice defect frequencies. The lengths of individual microtubules were measured on 21 selected good quality x8 binned tomograms of primary processes in Fiji. Good quality tomograms had the following characteristics: thin enough ice, minimal contamination, minimal distortions, good alignment, relatively high signal-to-noise ratio, and preservation of structural details. The number of MIPs and lattice defects in each microtubule were manually counted in 3dmod (version 5.1) using the slicer and the ZAP window (115). For each microtubule, the MIP and lattice defect frequencies were calculated by dividing their number by the microtubule length. The distance from the cell body to the primary process where the tomogram was acquired was estimated using Fiji in low resolution images of the area. Microtubules from tomograms of all the primary processes were pooled and categorized based on the distance from the cell body.

### Determination of filament abundance and lengths in astrocyte primary processes

Tomograms acquired on 10 different primary processes across 4 cells were used in this analysis. 11 good quality tomograms were used to analyze lengths and abundance of different classes of filaments. Of the 11 tomograms, 8 tomograms were used to generate data 10 – 20 μm, 5 at 0–10 μm, 2 at 20–30 μm, and 1 at 30–40 μm from the cell body. Like microtubules, lengths of individual IF and actin filaments in tomograms acquired at the primary processes were measured in Fiji. Frequency counts of each filament type were used to generate a percentage abundance of each filament class in the region of tomogram acquisition.

### Statistics

Means, standard deviations, standard errors, and statistical analyses of immunofluorescent data were calculated and graphed using GraphPad Prism 10 (Version 10.4.0 (527)). Data were tested for normality or parametricity using Shapiro-Wilk and Kolmogorov-Smirnov tests. Normal data were analyzed using t-tests or ANOVAs while non-normal data were analyzed with Mann-Whitney or Kruskal-Wallis tests. In ANOVA analyses, repeated measures were used since multiple measurements from cells cultured together cannot be considered completely independent from one another. Box plots for both MIP and lattice defect frequencies were plotted and statistical pairwise tests were performed in R (R Core Team. 2025).

## Supplementary Material

1
