# Supplementary material for: Spatially patterned cytoskeletal organization shapes astrocyte branch complexity
Source: bioRxiv. 2025 Oct 11:2025.10.10.681734. Preprint. [Version 1] doi: 10.1101/2025.10.10.681734 (PMC12632303; doi:10.1101/2025.10.10.681734)
Supplement: 1 [file NIHPP2025.10.10.681734v1-supplement-1.pdf]

bioRxiv preprint doi: https://doi.org/10.1101/2025.10.10.681734; this version posted October 12, 2025. The copyright holder for this preprint (which was not certified by peer review) is the author/funder, who has granted bioRxiv a license to display the preprint in perpetuity. It is made available under aCC-BY 4.0 International license.

Supplementary Figures and Legends

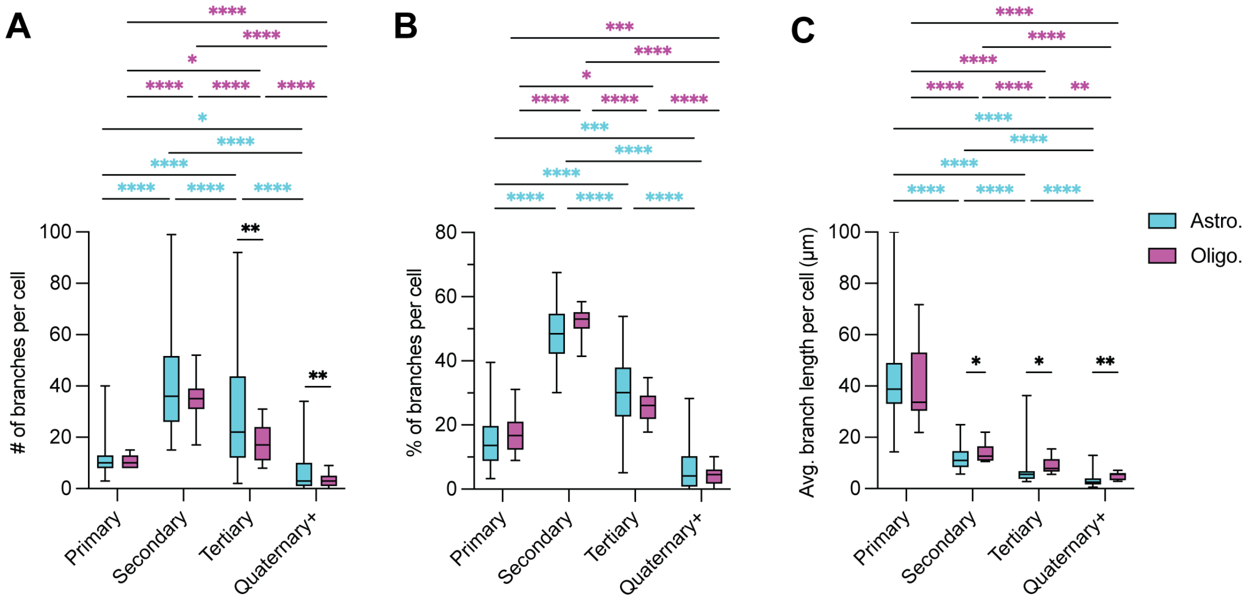

Fig. S1. Branch complexity in immunopanned astrocytes and oligodendrocytes.
(A–C) Box and whiskers plot quantifications of the number **(A)**, percentage **(B)**, and length (µm) **(C)** of primary, secondary, tertiary, and quaternary branches per cell in primary astrocytes and oligodendrocytes. Whiskers extend from minimum to maximum values and boxes extend from 25th to 75th percentiles with a line at the median. n = 15–44 cells from 3–4 independent cultures (from 3–4 litters of rats). 2-way ANOVA with repeated measures or mixed-effects model with repeated measures, followed by Tukey's multiple comparisons testing. * $p \leq 0.05$, *** $p \leq 0.001$, **** $p \leq 0.0001$. Black asterisks show comparisons between astrocytes and oligodendrocytes. Cyan asterisks compare branch order within astrocytes. Magenta asterisks compare branch order within oligodendrocytes. mean ± SEM for primary, secondary, tertiary, and quaternary categories from left to right: (A) Astro: 11.3 ± 0.9, 42.7 ± 3.3, 28.2 ± 3.2, 6.9 ± 1.3; Oligo: 10.7 ± 0.6, 34.5 ± 2.6, 17.6 ± 1.9, 2.9 ± 0.6, (B) Astro: 15.1 ± 1.2, 49.2 ± 1.5, 29.1 ± 1.6, 6.6 ± 1.1; Oligo: 17.5 ± 1.7, 52.3 ± 1.1, 26.1 ± 1.3, 4.1 ± 0.8, (C) Astro: 45.0 ± 3.4, 11.7 ± 0.7, 6.4 ± 0.8, 3.2 ± 0.4 µm; Oligo: 40.4 ± 3.8, 14.2 ± 1.0, 8.9 ± 0.8, 5.0 ± 0.5 µm.

bioRxiv preprint doi: https://doi.org/10.1101/2025.10.10.681734; this version posted October 12, 2025. The copyright holder for this preprint (which was not certified by peer review) is the author/funder, who has granted bioRxiv a license to display the preprint in perpetuity. It is made available under aCC-BY 4.0 International license.

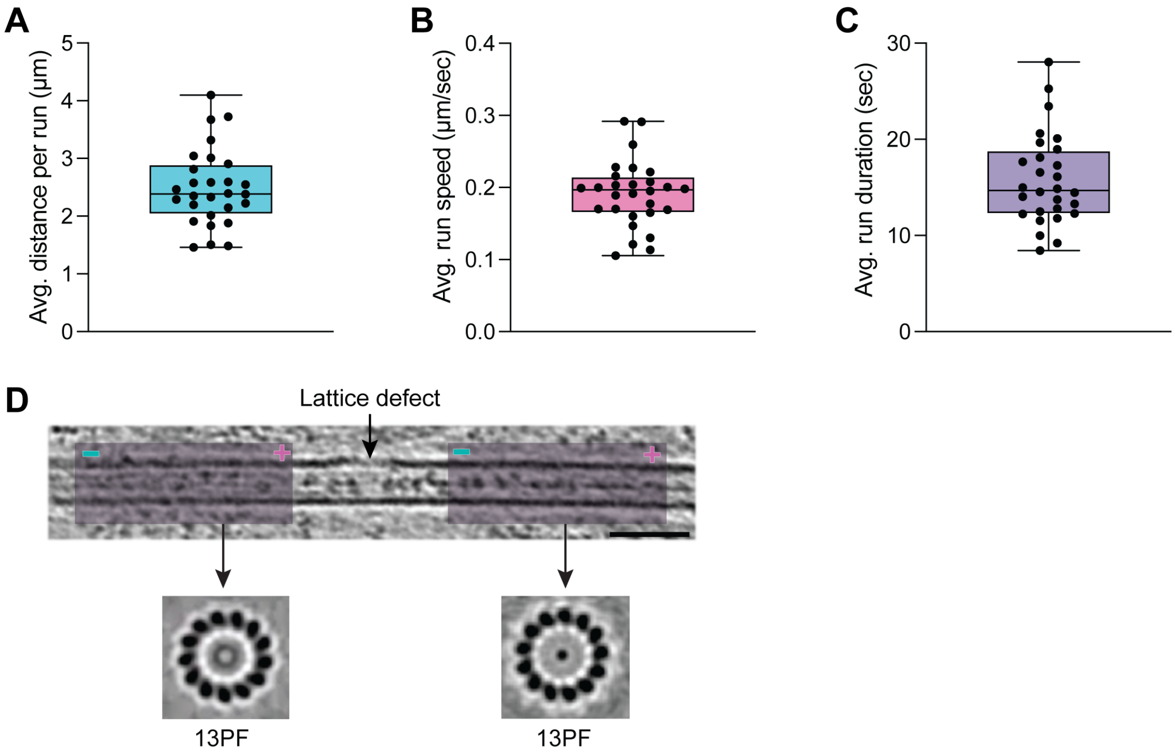

Fig. S2. Extended analysis of EB3 comet runs and maintenance of microtubule polarity and protofilament number in primary astrocytes.
(A–C) Box and whisker plots summarizing means per cell for run distance **(A)**, run speed **(B)**, and run duration **(C)** for EB3-mNeonGreen (EB3-mNG) comets in primary astrocytes expressing EB3-mNG. n = 110 kymographs from 28 cells from 3 independent cultures (3 litters of rats). **(D)** Tomogram slice containing a microtubule with a lattice defect (at the arrow, which points to missing protofilaments, as indicated by the lower gray level at the edge of the projection image). Below: end-on views of subtomogram averages showing maintenance of 13-protofilament geometry and plus-ends-out polarity before and after the break. Dark purple boxes correspond to the areas used for averaging. Only microtubules with consistent averages along the lattice were analyzed. Scale bar: 100 nm.

bioRxiv preprint doi: https://doi.org/10.1101/2025.10.10.681734; this version posted October 12, 2025. The copyright holder for this preprint (which was not certified by peer review) is the author/funder, who has granted bioRxiv a license to display the preprint in perpetuity. It is made available under aCC-BY 4.0 International license.

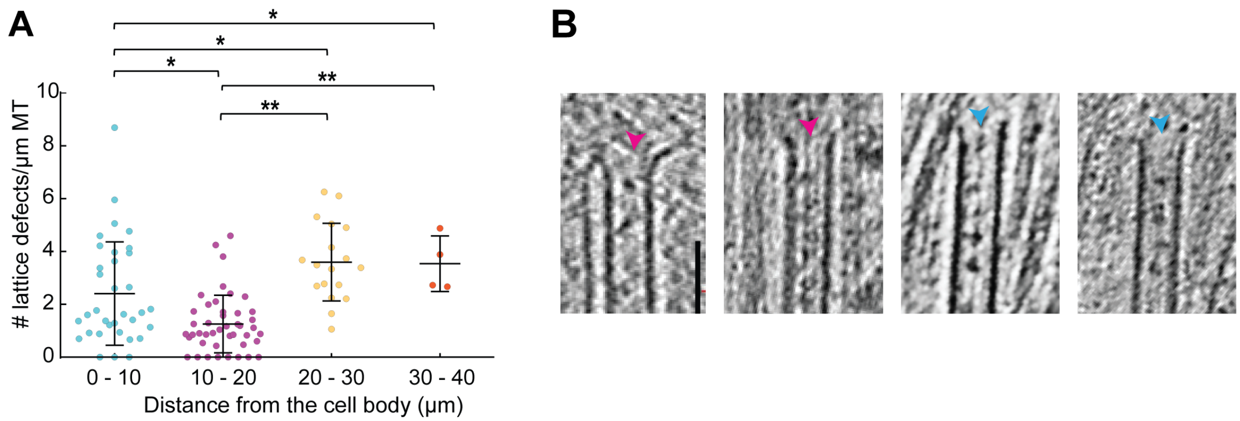

Fig. S3. Lattice defects and morphology of microtubule ends in astrocyte primary processes.

(A) Graph depicting the lattice defect frequency per µm microtubule length in astrocytic primary processes, binned by distance from the cell body: 0–10 µm (n = 35 microtubules), 10–20 µm (n = 46), 20–30 µm (n = 18), and 30–40 µm (n = 4). Bars represent mean ± SD. ** p < 0.01, by pairwise comparisons. **(B)** Representative tomogram examples of microtubule ends highlighting slightly flared (pink arrowheads) and blunt (blue arrowheads) ends. Scale bars: 100 nm.

bioRxiv preprint doi: https://doi.org/10.1101/2025.10.10.681734; this version posted October 12, 2025. The copyright holder for this preprint (which was not certified by peer review) is the author/funder, who has granted bioRxiv a license to display the preprint in perpetuity. It is made available under aCC-BY 4.0 International license.

A

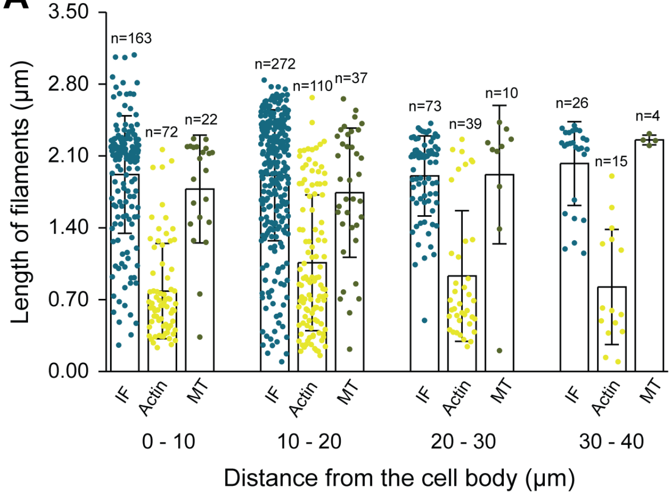

B

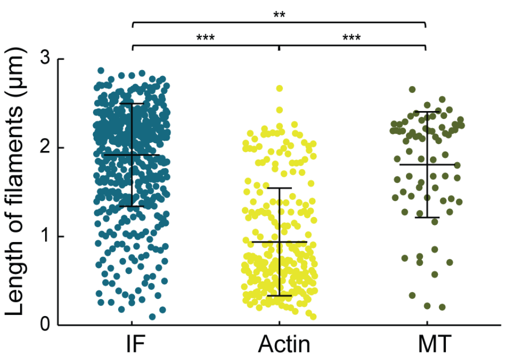

C

Filament Abundance (%)

	IF	MT	F-Actin
**0-10 µm**	76.6	9.6	13.8
**10-20 µm**	74.2	9.2	16.6
**20-30 µm**	71.5	9.9	18.7
**30-40 µm**	71.1	12.2	16.7

D

Filament Ratios

	IF:MT	Actin:MT	IF:Actin
**0-10 µm**	7.4	3.3	2.3
**10-20 µm**	7.4	3.0	2.5
**20-30 µm**	7.3	3.9	1.9
**30-40 µm**	6.5	3.8	1.7

E

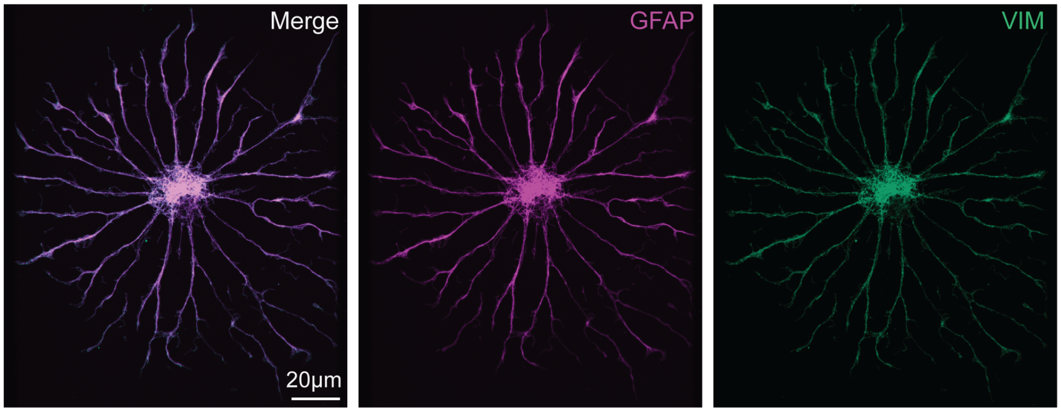

Fig. S4. Composition of filament classes in astrocyte primary processes and co-localization of GFAP and vimentin.
(A) Bar graph showing the lengths of different filament classes measured in tomograms acquired in astrocytic primary processes, binned by distance from the cell body: 0–10 µm, 10–20 µm, 20–30 µm, and 30–40 µm. n above each bar shows the number of individual filaments analyzed. **(B)** Bar graph showing average length of different filament classes in primary astrocyte processes, pooling all filaments together analyzed in (A). Bars in (A) and (B) represent mean ± SD. **(C)** Table depicting percentage abundance of each filament class in primary astrocyte processes, calculated with raw numbers of filaments in (A) and binned by distance from the cell body: 0–10 µm, 10–20 µm, 20–30 µm, and 30–40 µm. **(D)** Table showing ratios of different filament classes to one another in primary astrocyte processes, calculated with raw numbers of filaments in (A) and binned by distance from the cell body: 0–10 µm, 10–20 µm, 20–30 µm, and 30–40 µm. **(E)**

bioRxiv preprint doi: https://doi.org/10.1101/2025.10.10.681734; this version posted October 12, 2025. The copyright holder for this preprint (which was not certified by peer review) is the author/funder, who has granted bioRxiv a license to display the preprint in perpetuity. It is made available under aCC-BY 4.0 International license.

Representative immunofluorescence staining image of GFAP and vimentin in primary astrocytes, showing overlapping localization of these IFs.

bioRxiv preprint doi: https://doi.org/10.1101/2025.10.10.681734; this version posted October 12, 2025. The copyright holder for this preprint (which was not certified by peer review) is the author/funder, who has granted bioRxiv a license to display the preprint in perpetuity. It is made available under aCC-BY 4.0 International license.

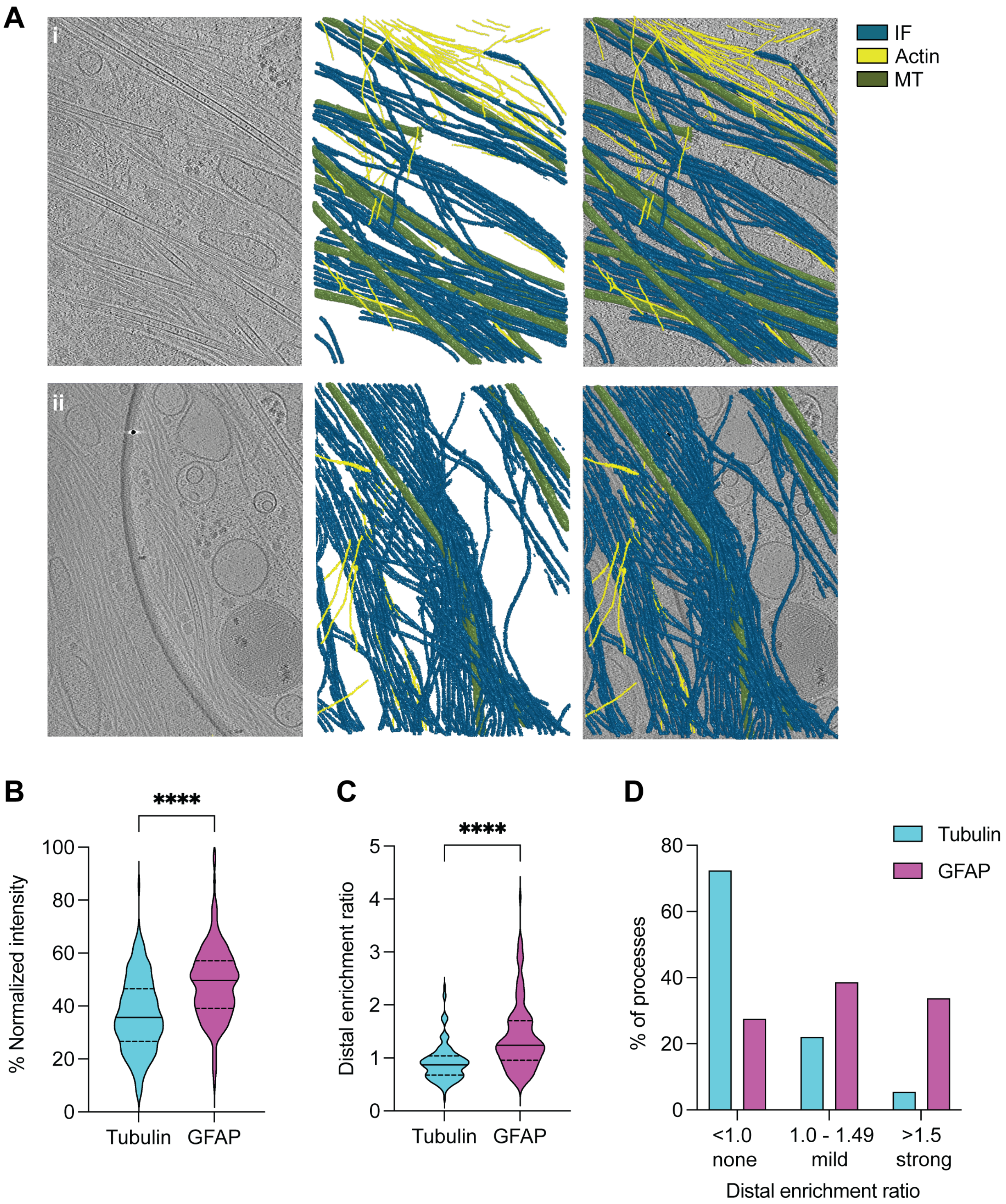

Fig. S5. Interplay between microtubules and IFs observed by cryo-ET and extended analysis of distal GFAP enrichment in astrocyte process tips.

(A) 2D slices of two example tomograms (i, ii) highlighting the presence of IFs alongside microtubules. In both i and ii, the rightmost image shows a 2D slice of a reconstructed and CTF-corrected tomogram, the middle image shows segmentation of cytoskeletal filaments based on neural-network training, and the left image shows segmentation overlaid on the tomogram. Scale bar: 50 nm. **(B)** Violin plot depicting the percent intensity of tubulin and GFAP across the distal 10

bioRxiv preprint doi: https://doi.org/10.1101/2025.10.10.681734; this version posted October 12, 2025. The copyright holder for this preprint (which was not certified by peer review) is the author/funder, who has granted bioRxiv a license to display the preprint in perpetuity. It is made available under aCC-BY 4.0 International license.

µm of primary and secondary astrocyte processes. Intensities were normalized against the maximum value across the entire branch. Mean ± SEM for tubulin and GFAP, respectively, are 36.8 ± 1.2% and 49.6% ± 1.1%. **(C)** Violin plots showing significantly more distal enrichment of GFAP compared to tubulin. Distal enrichment ratio was calculated as the average % normalized intensity in the distal 10 µm of process tips divided by the average % normalized intensity in the preceding 40 µm. Mean ± SEM for tubulin and GFAP, respectively, are 0.91 ± 0.03 and 1.40 ± 0.05. **(D)** Bar graph showing percentage of processes classified by variable distal enrichment ratios. Enrichment ratios below 1.0 correspond to lower intensity in the distal 10 µm. Mild enrichment ratios correspond to <50% higher intensity across the distal 10 µm. Strong distal enrichment ratios correspond to >50% higher intensity in the distal 10 µm. (B) and (C) analyzed with Mann-Whitney test, **** $p \leq 0.0001$. For (B–D), n = 141 processes from 25 cells across 3 independent experiments (from 3 litters of rats).

bioRxiv preprint doi: https://doi.org/10.1101/2025.10.10.681734; this version posted October 12, 2025. The copyright holder for this preprint (which was not certified by peer review) is the author/funder, who has granted bioRxiv a license to display the preprint in perpetuity. It is made available under aCC-BY 4.0 International license.

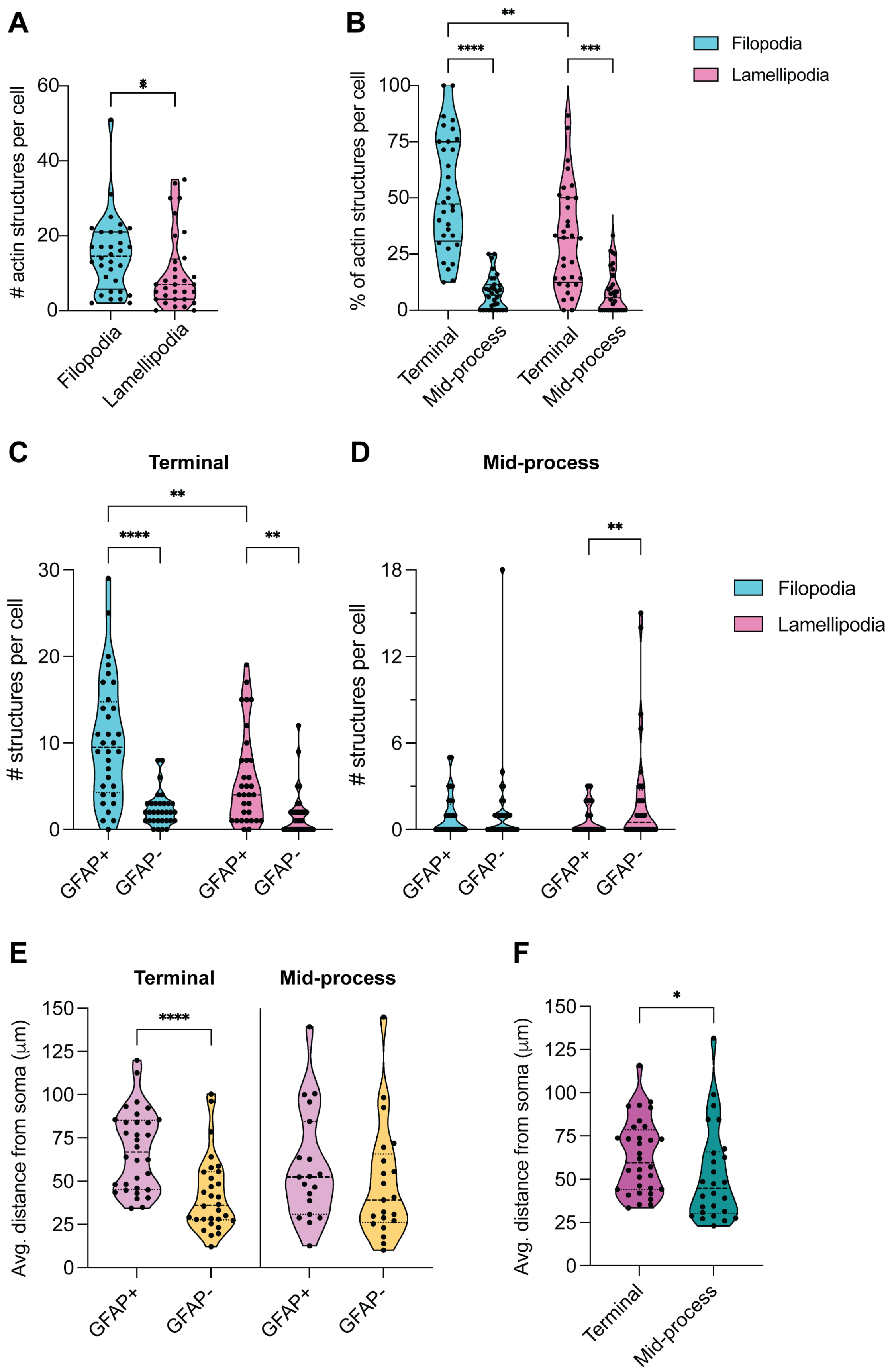

bioRxiv preprint doi: https://doi.org/10.1101/2025.10.10.681734; this version posted October 12, 2025. The copyright holder for this preprint (which was not certified by peer review) is the author/funder, who has granted bioRxiv a license to display the preprint in perpetuity. It is made available under aCC-BY 4.0 International license.

Fig. S6. Expanded analysis of actin microstructures and GFAP co-localization in astrocyte processes.
Violin plots comparing: **(A)** Total numbers of filopodia and lamellipodia per astrocyte. **(B)** Percentages of filopodia versus lamellipodia per astrocyte, segregated by terminal versus mid-process location. **(C, D)** Numbers of GFAP-positive versus GFAP-negative filopodia or lamellipodia per astrocyte, segregated by location. **(C)** In the terminal, GFAP-positive filopodia (mean ± SEM: 10.3 ± 1.3 per cell) significantly outnumbered GFAP-positive lamellipodia (5.8 ± 1.0); no difference was observed between the numbers of GFAP-negative filopodia (2.3 ± 0.4) and GFAP-negative lamellipodia (1.8 ± 0.5). **(D)** At mid-process sites, lamellipodia were more often GFAP-negative (2.1 ± 0.7) than GFAP-positive (0.7 ± 0.2). **(E, F)** Average distance per astrocyte from the edge of the soma to the actin microstructure, segregated by terminal versus mid-process location **(E),** or the presence or absence of GFAP **(F)**. Mann-Whitney test or 2-way ANOVA with repeated measures followed by Tukey's multiple comparisons testing. n = 33 cells from 4 independent cultures (from 4 litters of rats). * $p \le 0.05$, ** $p \le 0.01$, *** $p \le 0.001$, **** $p \le 0.0001$.

bioRxiv preprint doi: https://doi.org/10.1101/2025.10.10.681734; this version posted October 12, 2025. The copyright holder for this preprint (which was not certified by peer review) is the author/funder, who has granted bioRxiv a license to display the preprint in perpetuity. It is made available under aCC-BY 4.0 International license.

Movie S1.
Example live movie of astrocytes transfected with EB3-mNeonGreen to label growing microtubule plus ends. Movie was acquired using 3 second intervals over 3 minutes total. Framerate is 10 FPS.
